# Long-term Outcome with Paclitaxel Drug-coated Balloon in the Real World: Focus on Those Most at Risk

**DOI:** 10.1007/s00270-023-03556-1

**Published:** 2023-09-26

**Authors:** Ulf Teichgräber

**Affiliations:** Department of Diagnostic and Interventional Radiology, Jena University Hospital, Friedrich-Schiller-University Jena, Am Klinikum 1, 07747 Jena, Germany

The IN.PACT Global real-world study on the treatment of femoropopliteal peripheral artery disease with paclitaxel drug-coated balloon (DCB) angioplasty revealed sustained effectiveness and safety. Freedom from target lesion revascularization (TLR) at 5 years was 69% and freedom from 5-year all-cause mortality was 79% [1]. Whether long-term outcomes in high-risk participants with diabetes or chronic limb-threatening ischemia (CLTI) differ from those without has now been evaluated in a post hoc analysis. Results are presented in this issue of CVIR, by Reijnen et al. [2].

Results from the previous IN.PACT SFA randomized controlled trial (RCT) [3] have likely been basis for this research issue. In the total cohort, DCB was superior to standard balloon angioplasty (PTA) (freedom from TLR: 75 and 65%, respectively, *p* = 0.02). However, with diabetes, a treatment effect that had been demonstrated at 2 years (primary patency) was no longer evident at 5 years (freedom from TLR: 70 and 64%, respectively, *p* = 0.243). Whether the latter was due to limited power which remains open. The proportion of CLTI participants (about 5%) was too small for sufficient evidence on interaction. Nevertheless, Rutherford category did not interact with the treatment effect. At 5 years, no significant difference in all-cause mortality occurred between treatment groups. Probably due to the small number of events, no subgroup analysis on mortality regarding diabetes or CLTI was conducted.

The AcoArt I RCT provided another long-term evaluation of femoropopliteal DCB angioplasty [4]. In the total cohort that included more than half of participants with diabetes, 5-year freedom from TLR was 78% with DCB and 59% with PTA (*p* < 0.001), HR 0.42 (95%CI 0.24–071). Neither diabetes nor CLTI interacted with the treatment regarding freedom from TLR. There was no association of the treatment with all-cause mortality.Please provide complete details for references [3].Laird JA, Schneider PA, Jaff MR, Brodmann M, Zeller T, Metzger DC, Krishnan P, Scheinert D, Micari A, Wang H, Masters M, Tepe G. Long-Term Clinical Effectiveness of a Drug-Coated Balloon for the Treatment of Femoropopliteal Lesions. Circ Cardiovasc Interv. 2019 Jun;12(6):e007702. doi: 10.1161/CIRCINTERVENTIONS.118.007702. Epub 2019 Jun 14. PMID: 31195825; PMCID: PMC6636795.

The EffPac RCT [5] revealed a 5-year freedom from TLR of 82% after DCB and 74% after PTA (*p* = 0.05) and no difference between treatment groups concerning all-cause mortality. About one third of participants had diabetes. Subgroup analysis did not reveal an interaction effect of diabetes regarding primary patency throughout 5 years. The share of CLTI participants was negligible.

The recent post hoc comparison of effectiveness and safety of femoropopliteal DCB analyzed larger numbers of participants with diabetes and CLTI and therefore should be more reliable under this aspect. However, comparison groups were not randomized and thus results were prone to confounders. Unlike with subgroup analyses in RCTs, no interaction of subgroups (diabetes, CLTI) with the treatment could be assessed but rather the difference between diabetes and non-diabetes and CLTI and non-CLTI, respectively—not really the key issue. In addition, it should be emphasized that both diabetes and CLTI are particularly characterized by involvement of infra-popliteal lesions which represent a particular challenge. As the present study only considered femoropopliteal lesions, clinical significance and generalizability of the findings are considerably limited.

In accordance with previous RCTs on long-term effectiveness of femoropopliteal DCB, the study found no differences among participants with or without diabetes. All-cause mortality at 5 years was significantly higher with diabetes. Whether this would also be the case with PTA and whether there is any interaction with DCB, we cannot know from this work. Additionally, the analysis included 156 CLTI participants—considerably more than the above mentioned RCTs. As might be expected, 5-year freedom from TLR was lower and 5-year incidence of major amputation and all-cause death were higher in participants with CLTI compared to intermittent claudication. Again, no statement can be made on the impact of the treatment.

However, finally, one could conclude that femoropopliteal DCB angioplasty is efficacious in both patients with and without diabetes.
